# Health promotion and the randomised controlled trial: a square peg in a round hole?

**DOI:** 10.1186/1472-6831-9-1

**Published:** 2009-01-05

**Authors:** Ruth Freeman

**Affiliations:** 1 Oral Health and Health Research Programme, Dental Health Services Research Unit, University of Dundee, MacKenzie Bld, Kirsty Semple Way, Dundee, DD2 BF4, UK

## Abstract

In their paper published in BMC Oral Health in March, Barker and Horton present qualitative data which explored Latino parents' main concerns regarding accessing dental care for their pre-school children. In the radical discourse of health promotion the use of participant narratives is a first and essential step in community development interventions. While there is agreement regarding the development and implementation of health promotion, the means by which it is evaluated or the type of evaluation design used, is hotly debated. This commentary outlines the rationale of adopting a randomised controlled trial methodology, contrasts it with realistic evaluation and considers design evaluation in the light of the Medical Research Council's (MRC) guidance of 2000 and 2008. It is at this juncture that the commentary suggests that, despite the MRC's acknowledgement of the limitations of its 2000 guidance, there remains, in the 2008 guidance, an underlying insistence to use design evaluations which control for selection bias and confounding extraneous factors. For the evaluation of health promotion interventions it may remain a case of fitting a square peg into a round hole.

## Introduction

In their paper published in BMC Oral Health in March 2008, entitled 'An ethnographic study of Latino preschool children's oral health in rural California: intersections among family, community, provider and regulatory sectors', Barker and Horton [1] examined the barriers experienced by immigrant parents when accessing dental care for their pre-school children. The authors set out the main parental difficulties or concerns encountered. These included fears of official bodies and a lack of awareness of health care entitlements, where to find health care amenities and public transport routes. At a more personal level parents' concerns included; language differences and literacy problems; health professionals' lack of cultural sensitivity and how their children's dental fears would be managed. This cocktail of concerns, worries and anxieties resulted in parents delaying treatment visits and consequently increasing oral health disparities in this ethnic minority group. Why should Barker and Horton's [1] work be of importance: why should an in-depth examination or assessment of community concerns be of relevance? What is the relevance of participants' narratives, as an expression of their internal world, for the radical discourse of health promotion and its evaluation?

## Why assess the main concerns of participants?

In the debate surrounding the radical discourse of health promotion [2,3] it is acknowledged that 'community empowerment and advocacy are pivotal in the promotion and maintenance of health' [4] and that an appreciation of people's main concerns paves the way for empowerment. Thus people's narratives or their main concerns are central in the radical discourse of health promotion, together with the assurance that if health promotion interventions are to be successful then they must not exacerbate social isolation, increase self-deprecation or reduce self-esteem in those they wish to help. Nevertheless, health promotion interventions, which adopt the conventional discourse or a 'top-down approach', may unintentionally foist information which is thought to be of 'good' quality and content but is inappropriate to address people's health concerns. While such interventions may advance the health of those able to understand the health nuances; for those considered to be socially excluded with impoverished social networks, the consequence of a 'top-down approach' is to increase rather than decrease health inequality [5]. In contrast, the radical discourse adopts a 'bottom-up approach', in which people's concerns are centre-stage. Therefore whether people's main concerns are conceptualised in terms of narrative or 'tuning into the community's universe' [6] or as the 'core category' of grounded theory [7] – what is imperative is that the radical discourse or community development approach reflects the spoken narratives and unspoken concerns of the people. The importance of Barker and Horton's [1] work is that they used qualitative research methodologies. By doing so they enabled parents' voices and their health concerns to be heard – the first essential steps in the radical discourse of health promotion.

## 'Not just what works but why it works?'

In the usual course of events an intervention is implemented and randomised controlled trials (RCTs) are conducted in order to evaluate the success or otherwise of the intervention. While RCTs evaluate what works are they the most appropriate means of evaluating 'why the intervention works'[8,9]?

Central to the question of design evaluation is the ability of the researcher to control all extraneous factors which could result in selection bias. The problem is thought to be particularly acute in quasi-experimental designs where selection bias may cause contamination and distortion of intervention effects. The need for a non-biased selection of participants into experimental and control groups has been recognised as the way forward in the reliable and valid quest to discover 'what works' [9]. Randomisation procedures and standardized protocols are used to reduce selection bias. In the world of the RCT differences between experimental and control groups may be 'attributable to the intervention' while contamination is obliterated due to the scientific rigours of the protocol [9].

The requirement for standardised protocols for participant selection, randomisation and the reliance on 'uniform' [9] interventions to detect ever smaller intervention effects has resulted in time consuming and financially expensive multi-centred studies. Therefore while the RCT remains the design of choice for 'individualistic' or 'treatment' interventions, concerns have been raised by Pawson and Tilley [8] and Davies et al [9], as to its suitability and appropriateness when assessing 'social' or community development interventions [9]. Despite the rigours of the RCT methodology, it is considered by some to be inappropriate for the evaluation of community development interventions. This is particularly the case where extraneous environmental factors out-with the intervention and psycho-social factors internal to the participants may contaminate and dilute the intervention effect. The need to reassess the place of the RCT in health promotion evaluation is sorely needed.

Pawson and Tilley called for 'realistic evaluation' [8] while Davies et al stressed the need for 'theory-led' [9] assessments. It was apparent that in order to assess community development approaches the requirement to 'unpack the box' [8] or use 'within-programme experimentation' [9] meant that the use of qualitative methodologies such as grounded theory would be ideally placed to develop a theory [4,10] from which the 'why it works' could be evaluated. Therefore the within-programme evaluation would [i] 'find out why and for whom the programme might work'; [ii] conduct 'an outcome analysis internal to the programme on the context and mechanisms associated with success'; [iii] discover 'the participants' views and interpretations of the programme' [8]. Thus the requirement for ever greater restrictions in intervention evaluations had been questioned and the suitability of the RCT in the evaluation of community development interventions re-assessed.

## A square peg in a round hole?

How does realistic evaluation fit with the Medical Research Council's 'Framework for development and evaluation of RCTs for complex interventions to improve health'[11]? In 2000 the MRC provided a framework of 5 phases which, it could be argued, whittled away the richness of people's life experiences in order to enable complex interventions for health to be shoe-horned into RCTs. By stripping away the complexity of human interaction the MRC provided a framework bereft of compassion which bore little relationship to the richness of 'realistic evaluation' in the assessment of community development interventions.

On the 29^th ^September 2008, the MRC published 'Developing and evaluating complex interventions: new guidance'[12,13]. It included additional evaluation designs and with it 'identified limitations' [13] of the 2000 framework. In essence the appropriateness of the RCT as a 'one-size-fits-all' evaluation design was re-visited. There was, however, little appreciation that the rigidity of such design evaluations or a reliance on, for example, *à priori *theoretical models might act as confounders and sources of error in the evaluation process. Nevertheless and to quote Craig et al [13] 'many issues surrounding evaluation of complex interventions are still debated' and so the argument of the place of RCTs in the radical discourse of health promotion continues. While the MRC does not 'intend the revised guidance to be prescriptive' [13] it is perhaps with a sense of resignation that followers of a more radical discourse of health promotion may feel compelled to fit the square peg of community development assessment into the round hole of the RCT design evaluation.

## Pre-publication history

The pre-publication history for this paper can be accessed here:


